# Data utilisation and factors influencing the performance of the health management information system in Tanzania

**DOI:** 10.1186/s12913-021-06559-1

**Published:** 2021-05-25

**Authors:** Leonard E. G. Mboera, Susan F. Rumisha, Doris Mbata, Irene R. Mremi, Emanuel P. Lyimo, Catherine Joachim

**Affiliations:** 1 SACIDS Foundation for One Health, Sokoine University of Agriculture, P.O. Box 3297, Chuo Kikuu, Morogoro, Tanzania; 2 National Institute for Medical Research, Headquarters, Dar es Salaam, Tanzania; 3 Malaria Atlas Project, Geospatial Health and Development, Telethon Kids Institute, West Perth, Western Australia; 4 Ministry of Health, Community Development, Gender, Elderly and Children, Dodoma, Tanzania

**Keywords:** Health management information system, Data management, Data analysis, Utilisation, Health system performance, Factors, Tanzania

## Abstract

**Background:**

Health Management Information System (HMIS) is a set of data regularly collected at health care facilities to meet the needs of statistics on health services. This study aimed to determine the utilisation of HMIS data and factors influencing the health system’s performance at the district and primary health care facility levels in Tanzania.

**Methods:**

This cross-sectional study was carried out in 11 districts and involved 115 health care facilities in Tanzania. Data were collected using a semi-structured questionnaire administered to health workers at facility and district levels and documented using an observational checklist. Thematic content analysis approach was used to synthesise and triangulate the responses and observations to extract essential information.

**Results:**

A total of 93 healthcare facility workers and 13 district officials were interviewed. About two-thirds (60%) of the facility respondents reported using the HMIS data, while only five out of 13 district respondents (38.5%) reported analysing HMIS data routinely. The HMIS data were mainly used for comparing performance in terms of services coverage (53%), monitoring of disease trends over time (50%), and providing evidence for community health education and promotion programmes (55%). The majority (41.4%) of the facility’s personnel had not received any training on data management related to HMIS during the past 12 months prior to the survey. Less than half (42%) of the health facilities had received supervisory visits from the district office 3 months before this assessment. Nine district respondents (69.2%) reported systematically receiving feedback on the quality of their reports monthly and quarterly from higher authorities. Patient load was described to affect staff performance on data collection and management frequently.

**Conclusion:**

Inadequate analysis and poor data utilisation practices were common in most districts and health facilities in Tanzania. Inadequate human and financial resources, lack of incentives and supervision, and lack of standard operating procedures on data management were the significant challenges affecting the HMIS performance in Tanzania.

## Background

Data analysis and the use of information are essential components of a well-functioning health delivery system for planning and monitoring the progress of disease intervention programmes. In many low- and middle-income countries, the Health Management Information Systems (HMIS) have been established to enhance routine health facility-based data management [1]. The HMIS is expected to measure the magnitude of disease morbidity and mortality in populations, monitor trends over time, detect and hence facilitate prompt response to any unusual trends. In Tanzania, the HMIS is the primary information system, established during the early 1990s [2, 3]. It is composed of facility-based health records used for routine health services management, providing indicators for data on morbidity, mortality, health infrastructure, and service coverage. To strengthen the performance of HMIS in Tanzania, the Government adopted the District Health Information System (DHIS2), a web-based software package for collecting, validation, analysis, and presentation of aggregate statistical data tailored to integrated health information management activities. The adoption of DHIS2 aimed to facilitate data access and stimulate usage.

The effectiveness of a health information system depends on collecting, analysing, interpreting, and utilising the information correctly at all levels [4–6]. However, most often, incapacitated systems compromise optimal functioning and system performance.

Generally, in many Sub-Saharan African countries, data utilisation at all healthcare systems is low [4, 7–11]. Available literature suggests that, despite some notable successes, the impact of HMIS on the decision-making process within Africa health systems remains limited [4, 11, 12]. Several barriers have been reported to prevent the HMIS from achieving full potential in Africa [13, 14]. Institutional, technological, individual, and logistical capacities are perceived factors that either enable or impede the successful implementation and use of data generated from the HMIS [15]. It has been reported that though the HMIS offers opportunities to inform health decision-making at all levels of the health systems, its usefulness is realised only when it allows for the transformation of generated data into meaningful information and knowledge for action [7].

Routine health information is essential for both operational and strategic decision-making at all levels of the health system. Investment in health is dependent on efficient and reliable HMIS. With increased investment in disease control programmes in Tanzania, there is a critical need for a sound health information system to support decision-making at all levels and vice versa. It is envisaged that improved HMIS would enhance evidence-based decision and policy-making, leading to improved accountability and effectiveness at all health system levels. In the efforts to promote information utilisation and evidence-based decision-making, the Global Summit on Measurement and Accountability for Health has called for action for all countries to have health information flows involving utilising data locally to improve services effectiveness of disease programmes [16]*.* The objective of this study was to determine the utilisation of HMIS data and factors influencing the performance of the system at the district and primary health care facility levels in Tanzania.

## Methods

### Study sites and design

This cross-sectional study was carried out in November 2017 and involved 11 districts of Tanzania. It involved primary data collection at the health facility and district levels. For this study, the country was categorised into eight geographical zones. We used a multistage sampling technique to select the study regions and districts within the zones. Within zones, 1–3 regions were selected, and in each region, one district was selected randomly using a random generator function in M.S. Excel. Details of the study site selection have been described elsewhere [6].

### Sample size and data collection

Mixed methods employing both qualitative and quantitative techniques were used to gather the information needed for the study. Data were collected using a standard questionnaire and an observational checklist developed in consultation with experts in the field to ensure their content validity. Semi-structured guides for conducting in-depth interviews with health facility and district office staff were developed in English and translated into Kiswahili. The translated tools were pre-tested to assess their reliability before being adopted for the study. The questionnaire collected information on data management, including analysis and use, existing gaps, and challenges. The interviewees were asked how the data are analysed and used at their respective levels and feedback obtained from different levels. At this stage, observation and documentation of good practices on data management, analysis, and use, including displays available at a health facility or district offices, were done. Minutes from meetings were also reviewed to assess if data use is among the agenda in their routine discussions. The availability of dedicated staff responsible for managing data and health information systems, staff training on HMIS, and organisational and behavioural assessment availability was documented.

### Data management and analysis

Thematic content analysis was used to analyse the in-depth interviews. Briefly, the interviews were translated into English by the researchers. Then manual analysis was done by developing a code list from multiple readings of transcripts. This analysis was followed by open coding to identify major themes and sub-themes that emerged from the data, which were later related to the study objective. The observed practices on data analysis and use were summarised. Results obtained from both methods were triangulated for interpretation. In a case where quantification of results was needed, data were summarised into proportions.

## Results

### Socio-demographic characteristics of the respondents

This study involved 115 health facilities in 11 districts. A total of 93 health facility workers were interviewed. About two-thirds (65.6%) were Diploma holders. Clinical officers (43.0%) accounted for the majority of the respondents, followed by nurses (16.1%) and midwives (11.8%). Most (52.6%) of the respondents had over 10 years of working experience (Table 1). At the district office, 13 members of the Council Health Management Team were interviewed. They included District Medical Officers, District HMIS, and Reproductive Health Focal Persons.

**Table 1:** Socio-demographic characteristics of health facility respondents

Variable	Response	Frequency	Percentage
Sex	Male	45	48.3
	Female	48	51.6
Education qualification	Certificate	19	20.4
	Diploma	61	65.6
	Bachelor/Master degree	13	14.0
Experience (in years)	< 5	31	33.3
	5–10	13	13.9
	> 10	49	52.6
Position	Nurse	15	16.1
	Midwife	11	11.8
	Clinical officer	40	43.0
	Laboratory personnel	2	1.2
	Medical Attendant	4	4.3
	Medical Officer	10	9.8
Total		93	

### The utilisation of HMIS data at facility and district levels

#### Health facility level

Less than 10% (*n* = 11) of the facilities were found to conduct proper analysis and display data by plotting graphs that showed disease burden. About two-thirds of the facilities (60%) claimed to use the HMIS data they collect. Data were used for comparing performance in terms of services coverage (53%), determining morbidity and mortality trends over time (50%), providing public health education and promotion at their service areas (55%), and determining the facility requirements for drugs and other medical supplies (37%). The commonly identified types of data displayed at health facilities were top-ten diseases (58%). More than half (56%) of the facilities had displayed recent data analysis outputs (indicated to be of the past 3 months). However, most of the displayed information did not show dates; hence, detecting the respective period reported was difficult. Most facilities displayed top-10 diseases but only listing disease/conditions without any statistics to compare values. A variation in data processing and displays was observed between departments at health facilities. Most of the presentations were on reproductive and child health indicators and HIV/AIDS-related data.

Most respondents described the quality of data collected by their facilities to affect its utilisation in evidence-based decisions. They believed that decisions are based on the evidence or actual needs at both facility and district levels. These included the real needs of the service population and considering costs or the financial capacity of the facility. Furthermore, it was found that in some instances, decisions at health facilities were made based on directives from higher authorities. Few good practices on data use were claimed not to be noticed by superiors, and about two-third complained that staff was not rewarded for their excellent work but always made responsible for their poor performance. Regarding the importance of collecting health information, health facility workers strongly agreed that they understand why they must collect and submit data to appropriate levels and authorities. However, most of them agreed that collecting data that is not used for decision-making discourages them.

Nearly all (96.3%) of the health facilities reported having routine meetings to review managerial and administrative matters. Most of the health facilities (46.6%) reported that the meetings were held monthly, and nearly all (92.2%) maintained official records of the meetings. Furthermore, at least half (58%) of the facilities provided copies of proceedings of their previous meetings within the respective quarters. However, when the proceedings were examined, the majority (62.8%) did not have data as an agenda for discussions. Issues related to data that were observed from some of the proceedings included management of HMIS (data quality and reporting) (57%) and discussion about commodity stock-out (60%). Most of them (70.9%) had made decisions based on their discussion. Only about half (49.1%) of the facility reported participating in meetings convened by the district office to discuss HMIS data during the last 3 months.

#### District level

Data use at the district level was reported by more than half of the respondents (9/13). The district respondents reported using HMIS data mainly for monitoring and evaluating district targets. They reported that their districts produce reports that contain information from HMIS data. The commonly produced reports included: annual Council Comprehensive Health Plan, quarterly Council Health Management Team (CHMT) reports and Reproductive, and Child Health reports. Nine of the 13 district respondents reported systematically receive feedback on the quality of their summary HMIS reports from higher authorities. However, eight respondents reported that the district office does not provide feedback reports to the health facilities upon receiving the HMIS monthly reports.

Five out of 13 district respondents reported analysing data from the HMIS. A more significant proportion (10/13) of the respondents reported that the district office does not routinely hold meetings to review HMIS information, as illustrated by one respondent: *“We do not have routinely scheduled meetings hence proceedings of the meetings are not available. Our data are of poor quality because only a very few staff have received proper training on data management” (*Tandahimba*).* The three reported examples of promotion and use of reported data at the district level included public health education, annual budgeting, forecasting demand, and procurement of supplies. One of the district respondents said: “*The data help us budget and forecast on-demand and procurement” (Njombe).*

Districts were asked if the distribution of resources considers facilities that excel in HMIS performance. HMIS focal persons from 9 districts (Kinondoni, Tandahimba, Mbulu, Hai, Mbinga, Nkasi, Njombe, Igunga, and Dodoma urban) reported that priority is given to the allocation of resources to facilities with good performance. Only two districts (Kahama, Kibaha) reported not considering facility performance in the allocation of resources. Other criteria used for resource allocation in the annual plans included the size of the facility, the number of clients served, the number of services offered, disease burden, geographical location, and service priorities. However, only Kinondoni and Igunga districts were able to provide performance improvement tools such as flow charts and control charts to monitor the performance of their facilities. More than half (7/11) of the districts reported having a mechanism for generating funds for HMIS. Half of the respondents reported having a long-term financial plan for supporting HMIS activities, while the other half reported having no such plans.

### Factors influencing HMIS performance

#### Inadequate human, financial resources, and training

The number of staffs specifically responsible for HMIS activities differed from one district to another. All the facility respondents reported on the inadequacy of human resources for data management. The majority of those responsible for HMIS activities reported having received short training on data management, focusing only on proper recording, data collection, and reports with very elementary analysis or interpretation. However, few claimed that no specific training had been provided to them except for on-job training. A relatively large proportion (41.4%) of the facility’s personnel had not received any training on data management related to HMIS 12 months prior to this survey. On average, there were five staff involved in HMIS activities per facility - from recording - a compilation of reports, while about 21 staff were trained during the previous 12 months (Table 2). The five staff who were reported to be directly involved in HMIS had to attend other activities too. With this few staff engaged in the HMIS activities, data management was reported to be compromised. One main challenge emphasised by district key informants was limited human capacity in HMIS to apply the analytical tools and methods to synthesise information for decision-making. This inefficiency was attributed to inadequate training. Only a few (3/11) reported having district training plans. Lack of financial resources was attributed to insufficient in-service training of staff on data management. At the district, all HMIS focal persons reported to have received training and provided with HMIS training manuals for reference on how to conduct HMIS-related tasks. More than half of district respondents (*n* = 7) reported that those responsible for HMIS activities were also responsible for other activities.

**Table 2:** Status of district staff who received training of on new HMIS tools

District	No. of staff involved HMIS activities	No. staff trained
Kibaha	7	1
Kinondoni	4	70
Tandahimba	2	1
Mbulu	3	1
Mbinga	5	2
Nkasi	9	0
Njombe	4	30
Kahama	3	40
Dodoma	7	19
Igunga	5	27
Hai	8	37
Average	5.2	20.7

#### Supervision

Less than half (42%) of the health facilities reported that members of the district office provided supportive supervisory visits to the facilities during the 3 months before this assessment. However, over half (52.4%) reported that the district supervision team did not have a supervision checklist during their visit. The majority of the facility respondents (72.1%) reported that the district team had conducted an audit of their data quality. Two-thirds of the facility respondents reported that the district team discussed the performance of the health facility based on the data provided, whereas only a few (9.6%) of the health facilities reported having an explicit schedule for district supervisory visits. Some respondents (37.5%) explained that district teams often pay visits without informing the respective facilities. Less than half of the respondents (44.9%) provided the district supervisory reports (Table 3). Contrary to the facility report, 9/11 districts reported having checklists and visit schedules, and 10/11 districts had supervisory reports.

**Table 3:** Health workers’ responses on the availability of tools needed during district supportive supervision

Item	Response	No. of respondents	Percentage
CHMT supervisory checklist	Yes	50	47.6
	No	55	52.4
	Total	105	
Schedule for CHMT visits	Yes	10	9.6
	No	94	90.4
	Total	104	
CHMT supervisory report	Yes	45	44.9
	No	57	55.9
	Total	102	

Key: *CHMT* Council Health Management Team

#### Standard procedures and HMIS tools for data management

The use of different procedures and logs for receiving reports was typical and associated with the loss of transmitted reports from lower levels. Some districts did not have a proper mechanism of receiving reports from health facilities. In such districts, any person who happened to be present at the district office when a report is delivered could receive the report without verifying proper filling or even documenting/acknowledging its receipts. Feedback on reporting and quality of reports from the district office to health facility workers was mentioned by only 54% of respondents, mainly verbal feedback. Such practices and communication gaps provided room for the incorrectly/poorly filled and incomplete reports from the facility level to be received by the district. During interviews with district officials, almost all districts (90%) reported that there were no written procedures in place to address late, incomplete, inaccurate, and missing reports, including follow-up with the facility on data quality issues, as one respondent said: *“There were no written procedures, just verbal discussions were provided some times through phone calls”* (Kinondoni)*.*

Registers, tally sheets, or report forms with no identification of health facility, or the reporting period, were common. Only six facilities were found to have logbooks available for marking and reporting submitted reports. At the same time, only two districts were observed to have stamped all the facility forms to indicate the receiving date. Only half of the districts reported having had quality control in place for data entry from paper-based to computer DHIS2. Different facilities in various districts used different versions of HMIS tools. Some of the versions did not have the variables of interest.

## Discussion

Generally, the utilisation of health data collected from health facilities in Tanzania is relatively poor. About two-thirds of the facilities reported using the HMIS data they collect, mainly comparing performance between service coverage, determining disease trends over time, and community health education and promotion. However, at the district level, the primary use of HMIS was for preparing reports and annual planning. This use suggests the underutilisation of all important data collected from health facilities. Several studies have identified weaknesses in data use and response in Tanzania while recognising that these are critical components for sound public health decision-making [4, 12, 17–20]. A similar low utilisation of routine facility data has been reported from elsewhere in Sub-Saharan Africa. For example, in a study in Ethiopia, training, data analysis skills, supervision, regular feedback, and favourable attitude were factors related to routine health information system utilisation [21]. The findings of this study and others elsewhere indicate that data is collected for reporting purposes, and there is minimal utilisation of the information to inform decisions. Usually, to most health workers in low-income countries, HMIS is associated with the filling of registers, compiling, and submitting reports to the next level without its utilisation [22, 23].

Studies elsewhere have reported that data generated by health facilities are most often not sufficiently utilised to improve health care [24–26]. Generally, in Sub-Saharan Africa, health information utilisation at primary health care and the district is poor [22]. In Ethiopia, the level of HMIS data utilisation for different decision-making purposes has been reported at 57.9–62.7% [27–29]. In a recent study in Zanzibar, it was reported that only 42% of the healthcare workers used HMIS data for monitoring and evaluation, 35% for planning, 23% for supply and drugs management, 18% for budgeting, and 10% for disease outbreak preparedness [30]. Factors associated with good utilisation of HMIS data have been described to include staff motivation, training, supportive supervision, a good perceived culture of health information, competence, and decisions based on superior directives [28, 29].

Several factors, including a limited number of staff and skills, low motivation, inadequate resources, lack of training and refresher courses, combined with lack of incentives and tools, have been pointed out to be responsible for the underperformance of HMIS in low- and middle-income countries [13, 31–34]. This study observed inadequate human resource for data management in all health facilities. In a study in Ethiopia, the availability of a standard set of indicators, skilled human resources, well-designed reporting formats, and staff trained to fill formats increased the likelihood of achieving data quality [35]. Organisational factors such as the culture of using information, resource availability, planning, governance, training, supportive supervision, and availability of finances have been reported to influence HMIS performance [36]. In Tanzania, Simba and Mwangu [19] found that trained staff on HMIS and seeking or provision of frequent feedback were significantly associated with performance.

In a recent study in Kenya, there was a significant positive relationship between the availability of adequate staffing for HMIS tasks, training of staffs, supervision of HMIS activities, availability of plans for HMIS, promotion of a culture of information, staff motivation, and the performance of routine health information system [37]. In our current study, less than half of the health facilities reported that the district team members visited their facility for supervision. However, when made supervision visits, the team did not have a supervision checklist. Already health workers’ data analysis skills, feedback, and regular supervision have been described to affect the successful implementation and use of routine health information systems [21, 35, 38, 39]. A study in Uganda reported that self-efficacy and the presence of RHIS staff directly influence the use of HMIS data [40].

Moreover, it has been reported that adequate supportive supervision and health facility performance review to be significantly associated with good performance in HMIS [41]. Lack of standard operating procedures (SOPs) was reported as a barrier to health workers’ performance in data management. SOPs for data management at the health facility level have been shown to help improve HMIS data quality in Rwanda [42].

In this study, the majority of the HMIS focal persons were non-health information technicians. Lack of staff with core competence in data management and analysis is one of the core weaknesses identified to affect data performance in several studies [6, 20, 32]. Similar findings have been reported in other studies elsewhere [43]. It is high time for the Government of Tanzania to establish an appropriate carder for data analysis and define clear responsibilities at all levels. Thus, there is a need for a proper team of skilled and competent people at all levels. For the HMIS to work effectively and efficiently, there must be consistency and integrity between the human, supplies, and process aspects.

Despite the significant relevance of the findings obtained from this study, its design attempted to control various biases by ensuring representation of districts and facilities and improving the validity of findings by intertwined interviews with actual observations. It faces several limitations. First, its cross-sectional nature limits the generalisation of results into other time points. Some of the responses were time-bound, resulting in different findings if a similar study was done at different time points. Second, on the same note, the findings may only be relevant for Tanzania and countries with very similar functionality to the HMIS.

Similarly, some of the responses are based on the interviewees’ experiences. Therefore, those found at the time of this assessment contributed significantly to the nature of the results. Finally, in the context that health workers are not randomly placed, variations might be expected with a different set of respondents.

## Conclusions

Poor data utilisation was common in most of the districts and health facilities in Tanzania. Data management skills, inadequate supervision and feedback, inadequate resources, and inadequate capacity building were factors related to the routine performance of the health management information system in Tanzania. The study, therefore, recommends that there is a need to have adequate staffing who are trained in HMIS tasks and have clear governance structures for the system that are communicated to all the health workers. In addition, there is a need to conduct periodic support supervision for HMIS activities, use innovative ways to motivate staff to perform HMIS related tasks and use evidence from routine health data to make decisions.

## Data Availability

The data of this study are presented in the main manuscript. Any additional supporting files available from the corresponding author on reasonable request.

## Abbreviations

CHMT: Council Health Management Team
DHIS: District Health Information System
HFR: Health Facility Register
HMIS: Health Management Information System
